# Psychometric evaluation of a parent-rating and self-rating inventory for pediatric obsessive-compulsive disorder: German OCD Inventory for Children and Adolescents (OCD-CA)

**DOI:** 10.1186/s13034-019-0286-z

**Published:** 2019-06-18

**Authors:** Julia Adam, Hildegard Goletz, Svenja-Kristin Mattausch, Julia Plück, Manfred Döpfner

**Affiliations:** 1 School of Child and Adolescent Cognitive Behavior Therapy at the University Hospital Cologne, Pohligstr. 9, 50969 Cologne, Germany; 2 Department of Child and Adolescent Psychiatry, Psychosomatics and Psychotherapy, Medical Faculty of the University of Cologne, Robert-Koch-Str. 10, 50931 Cologne, Germany

**Keywords:** Obsessive-compulsive disorder, Children, Adolescents, Assessment, Reliability, Validity

## Abstract

**Background:**

This study assesses the psychometric properties of the German version of the Padua Inventory-Washington State University Revision for measuring pediatric OCD.

**Methods:**

The parent-rating and self-rating inventory is assessed in a clinical sample (CLIN: n = 342, age range = 6–18 years) comprising an OCD subsample (OCDS: n = 181) and a non-OCD clinical subsample (non-OCD: n = 161), and in a community sample (COS: n = 367, age range = 11–18 years).

**Results:**

An exploratory factor analysis yielded a four-factor solution: (1) Contamination & Washing, (2) Catastrophes & Injuries, (3) Checking, and (4) Ordering & Repeating. Internal consistencies of the respective scales were acceptable to excellent across all samples, with the exception of the self-report subscale Ordering and Repeating in the community sample. The subscales correlated highly with the total score. Intercorrelations between the subscales were mainly r ≤ .70, indicating that the subscales were sufficiently independent of each other. Convergent and divergent validity was supported. Participants in the OCD subsample scored significantly higher than those in the non-OCD clinical subsample and the COS on all scales. In the COS, self-rating scores were significantly higher than parent-rating scores on all scales, while significant mean differences between informants were only found on two subscales in the OCD subsample.

**Conclusion:**

The German version of the Padua Inventory-Washington State University Revision for measuring pediatric OCD is a promising, valid and reliable instrument to assess self-rated and parent-rated pediatric OCD symptoms in clinical and non-clinical (community) populations.

**Electronic supplementary material:**

The online version of this article (10.1186/s13034-019-0286-z) contains supplementary material, which is available to authorized users.

## Background

Obsessive-compulsive disorder (OCD) is a severe mental disorder, characterized by obsessions, compulsive rituals, or both. Its prevalence rate in childhood and adolescence lies at approximately 1 to 4% [1, 2], and up to half of adult patients diagnosed with OCD report an onset of the disorder during childhood or adolescence [3]. To identify symptoms and treat the disorder as early as possible, appropriate assessment instruments for pediatric OCD are needed. OCD symptoms lead to a high psychological strain, distress and psychosocial impairment in children and adolescents [4], and considerably interfere with quality of life [5]. These serious consequences of the disorder have encouraged clinicians and researchers to develop new assessment instruments [6].

Several pediatric OCD-specific measures have been developed, which assess the self-report of children and adolescents only [7–10]. Most of these measures showed satisfactory internal consistencies and there is at least some support for their convergent and/or divergent validity. However, there is a need to assess OCD symptoms as rated by parents and children separately, because younger children may be unable to report their OCD symptoms accurately. Moreover, some children and adolescents may not report their symptoms accurately due to shame and embarrassment about their OCD [11]. On the other hand, parent reports may give underestimations because some symptoms (e.g. recurrent thoughts) are more difficult for parents to notice [12].

Overall, correlations between parent ratings and self-ratings have usually been found to be low, both in the assessment of mental health problems in children and adolescents generally (e.g. [13]) and in the assessment of OCD symptoms in particular [11]. Thus, to achieve a comprehensive clinical picture of the disorder, a multiple-informant assessment is required.

Therefore, researchers have recently developed questionnaires which encompass both self- and parent reports (*child*-*report version and parent*-*report version of the CY*-*BOCS,* CY-BOCS-CR, CY-BOCS-PR [14]; *Children’s Obsessional Compulsive Inventory,* CHOCI/CHOCI-R [15, 16]. Satisfactory internal consistencies have predominantly been reported for these questionnaires. However, analyses in a community sample revealed poor internal consistency for the Obsession and the Compulsion subscales and the Total scale of the CY-BOCS-CR [17]. Support for convergent and/or divergent validity was found for both instruments. However, only global scores for OCD symptoms or obsessive symptoms and compulsive symptoms were derived from these rating scales, while scales assessing different domains (e.g. controlling, washing) are not provided. This is also true for the only self- and parent-rated instrument developed for the German-speaking countries—the SBB-ZWA (Selbstbeurteilungsbogen für Zwangsspektrum-Störungen and the FBB-ZWA (Fremdbeurteilungsbogen für Zwangsspektrum-Störungen) [18].

Overall, none of these self-rated or parent-rated scales fulfill the criteria for a well-established assessment tool according to the criteria for evidence-based assessment (EBA; i.e.: reliability and validity must have been presented in at least two peer-reviewed articles by different investigators [19, 20]. Currently, the clinician-rated Children’s Yale-Brown Obsessive-Compulsive Scale (CY-BOCS [21]) is the only pediatric OCD-specific measure that can be classified as a well-established assessment according to these criteria [22].

In sum, despite the variety of self-report and parent-report forms for the assessment of pediatric OCD symptoms and severity/impairment, there is, to the best of our knowledge, only one measure, the Obsessive Compulsive Inventory-Child Version (OCI-CV) [7], that focuses on symptom frequency across symptom domains. However, The OCI-CV only exists in a self-report form. Clearly, there is a lack of instruments assessing symptoms across common OCD domains, and there are no measures that record both self- and parent report regarding OCD symptom domains. To gain a comprehensive clinical picture of the child or adolescent, however, the assessment should encompass multiple informants and perspectives.

Therefore, the current study examined an inventory to assess OCD symptoms in children and adolescents across common OCD domains, the OCD-CA (OCD Inventory for Children and Adolescents) [23], which is rated by children and parents separately and is based on the Padua Inventory-Washington State University Revision [24].

The main goals of the study are to: (1) identify the factor structure of the self-report and the parent-report form of the OCD-CA, (2) assess internal consistency of the subscales and the Total scale derived from factor analyses, (3) assess the correlations between the subscales for each informant, (4) assess the correlations between parent ratings and self-ratings, and (5) evaluate convergent and divergent and discriminant validity of the scales.

## Methods

### Instruments

The *German OCD Inventory for Children and Adolescents* (OCD-CA; German: Zwangsinventar für Kinder und Jugendliche; ZWIK [23]) is a modified version of the Padua Inventory-Washington State University Revision (PI-WSUR [24] /PI-WSUR (German translation) [25]). The OCD-CA enables the assessment of pediatric OCD symptoms on different symptom scales. The inventory comprises two multidimensional questionnaires: a parent form (target group: parents/caregivers of children and adolescents aged 6;0–18;11 years) and a self-report form (target group: children and adolescents aged 11;0–18;11 years), which are constructed analogously to one another. Accordingly, both rating forms include the same 36 items assessing various obsessions and compulsions. Parents or children/adolescents are asked to rate each item on a 5-point scale from 0 (not at all) to 4 (very much).

**Fig. 1:**
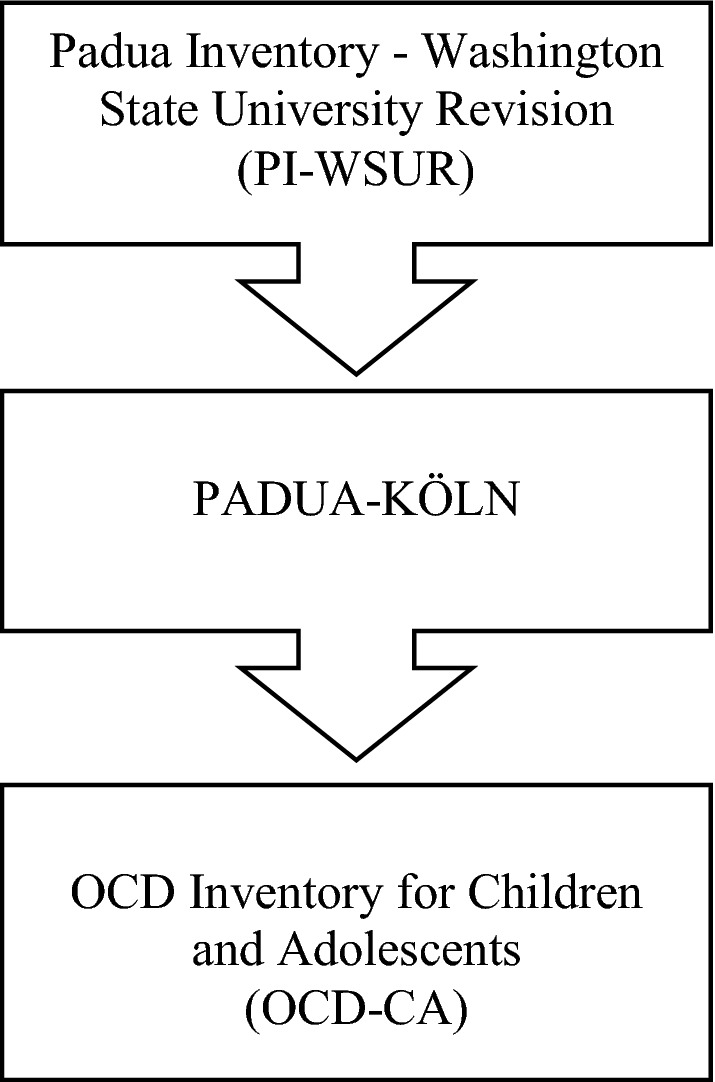
Development of the OCD-Inventory for Children and Adolescents

The development of the inventory is described below (see Fig. 1).

The starting point for the development was the revised version of the Padua Inventory [26–31], the *Padua Inventory*-*Washington State University Revision* (*PI*-*WSUR;* [24]). The PI-WSUR is a self-report measure assessing obsessions and compulsions in adulthood (applicable from the age of 16 years onwards). The instrument includes 39 items, rated on a 5-point scale from 0 (not at all) to 4 (very much) and measuring five OCD-relevant content dimensions: obsessional thoughts about harm to oneself or others, obsessional impulses to harm oneself or others, contamination obsessions and washing compulsions, checking compulsions, and dressing/grooming compulsions. As the PI-WSUR was found to be a valid and reliable questionnaire for the assessment of OCD symptoms in adulthood [24], the German translation of this instrument [25] was used as the basis for the development of the OCD-CA.

To compile a child-appropriate version, items of the PI-WSUR were transformed and extended concerning the most frequently occurring OCD symptoms in childhood. The item pool was developed through intensive discussion within a group of experienced clinical psychologists. Finally, thirty-two items of the German translation of the PI-WSUR were adopted and, in part, slightly changed to make items more suitable for children. For example, the PI-WSUR Item 1 “I feel my hands are dirty when I touch money” was changed to “I feel my hands are dirty when I touch money, books or toys”, and the PI-WSUR Item 18 “I keep on checking forms, documents, checks, etc., in detail to make sure I have filled them in correctly” was changed to “I keep on checking homework and other documents in detail to make sure I have completed them in correctly”. Seven items of the PI-WSUR were not adopted because they were assessed as not up-to-date or as not child-appropriate (e.g. Item 6 “I avoid using public telephones because I am afraid of contagion and disease” or Item 34 “While driving, I sometimes feel an impulse to drive the car into someone or something”). Furthermore, ten items were newly developed, which refer to repeating compulsions, counting, reassurance-seeking, (un)lucky number, hoarding/saving and not getting ready.

Accordingly, the first draft of a child-appropriate self-rating measure included 42 items assessed on a 5-point Likert scale, equivalent to the adult version. Analogously to the self-report form, a parent-report form was developed, including the same items. The self- and parent-report form were named *PADUA*-*KÖLN*. The PADUA-Köln was evaluated within a pilot study in a clinical sample (n = 55, age range 10–17 years). The adopted initial scale of the PI-WSUR *Obsessional Impulses to harm oneself or others* could not be confirmed through reliability analyses and comparison of means. Besides unsatisfactory internal consistency, comparisons of means showed that patients without OCD, especially those diagnosed with hyperkinetic disorders, had significantly higher means (self-reported and parent-reported) than patients affected by OCD. As a consequence, the PADUA-Köln was revised by eliminating the corresponding six items of the mentioned scale. The new scale was finally named *OCD Inventory for Children and Adolescents* (*OCD*-*CA*) (German*: Zwangsinventar für Kinder und Jugendliche; ZWIK*).

First analyses with the OCD-CA were conducted within a community sample (Waclawiak 2006, unpublished) comprising 367 self-reports and 434 parent reports (271 mothers and 163 fathers). Exploratory principal component analyses with varimax rotation (40 patients with OCD were included in the dataset to increase the variance in the sample) yielded a four-factor solution (Additional file 1). Internal consistencies for the self-report form and parent-report form (rated by mothers or fathers), respectively, were satisfactory to excellent for all subscales: Contamination Obsessions and Washing Compulsions (.86 ≤  α  ≤ .93), Checking and Repeating Compulsions (.82  ≤ α  ≤ .85), Obsessions concerning harm and injuries of others or oneself (.75 ≤ α  ≤ .78), Counting Compulsions and Reassurance-Seeking Compulsions and (un)lucky numbers (.77  ≤ α ≤ .85).

The German version of the *Children’s Yale*-*Brown Obsessive*-*Compulsive Scale* (*CY*-*BOCS*-*D* [32]) is based on the English original version of the CY-BOCS, developed by Goodman and colleagues (1986, unpublished scale). The clinician-rated CY-BOCS-D (based on parent/patient interview) comprises a symptom checklist and a semi-structured rating scale. The 58-item symptom checklist serves to assess the presence or absence of a variety of obsessions and compulsions. Symptoms can be summarized into four symptom scales [(1) obsessions regarding loss of control and religion; (2) checking, harm avoidance and sexual obsessions; (3) contamination and cleaning; (4) repeating, ordering/arranging, hoarding and magical thinking] and a total score. The 19-item rating scale serves especially to measure obsession severity, compulsion severity and the total OCD severity as well as to assess OCD-associated (personality) traits and abnormalities.

The OCD severity scale is derived by summing up the responses to the items 1–10, including items 1b and 6b. Items are rated on a 5-point Likert scale ranging from 0 to 4, with higher scores indicating greater symptom severity.

Psychometric evaluations of the CY-BOCS revealed positive results (see “Background”). The CY-BOCS-D symptom checklist and the rating scale displayed acceptable and good internal consistency, respectively. There was also evidence for the validity of the CY-BOCS-D [32]. In the present analyses, the symptom checklist scales and the total OCD severity score of the rating scale were used. Data were collected based on an interview with children and adolescents ≥ 11 years old with an OCD diagnosis (OCD subsample, see below).

The German version of the *Child Behavior Checklist*—*CBCL/6*-*18R* [33, 34], originally developed by Achenbach [35], is a parent-report instrument including 113 items which assess a range of behavioral and emotional problems in children and adolescents rated on a 3-point scale (“0 = not true”, “1 = somewhat or sometimes true”, “2 = very true or often true”). Items are assigned to two broad-band syndrome scales (Externalizing and Internalizing Problems) and eight syndrome scales. The German version shows good reliability and factorial validity [33, 34]. In the present study, the raw scale scores of the Internalizing and Externalizing scales were used.

The German version of the *Youth Self Report*—*YSR/11*-*18R* [34, 36], originally developed by Achenbach [37], is the equivalent self-report form of the CBCL (described above). The 112-item measure is child/adolescent-based and includes widely identical items to the CBCL. The structure and scales are the same. Research has also demonstrated good reliability (internal consistency) and factorial validity for the German version of the YSR [34, 36]. In the present study, the raw scale scores of the Internalizing and Externalizing scales were used.

The German *Symptom Checklists for Anxiety Disorders and Obsessive*-*Compulsive Disorders* are rated by parents (FBB-ANZ) of patients aged 6 to 18 years and by patients aged 11 to 18 years (SBB-ANZ). These scales are part of the Diagnostic System for the Assessment of Mental Disorders in Children and Adolescents based on the ICD-10 and DSM-IV (DISYPS-II) [38]. All items are rated on a 4-point Likert scale ranging from 0 (“not at all”) to 3 (“very much”). The questionnaires comprise 31 items describing anxiety symptoms and two items describing obsession and compulsion (scales: Separation Anxiety, Generalized Anxiety, Social Phobias, Specific Phobias and Total Scale). Psychometric evaluations of the SBB-/FBB-ANZ have yielded good results regarding reliability and validity [38]. The present analyses included the total score of the parent- and self-rated questionnaire.

The German *Symptom Checklists for Depressive Disorders* are likewise rated by parents (FBB-DES) of patients aged 6 to 18 years and by patients aged 11 to 18 years (SBB-DES). The rating scales are also part of the Diagnostic System for the Assessment of Mental Disorders in Children and Adolescents based on the ICD-10 and DSM-IV [38]. The structure, implementation and assessment are the same as described for the SBB-/FBB-ANZ. The total score includes 29 items. Psychometric evaluations of the SBB-/FBB-DES have also shown good results regarding reliability and validity [38]. Parent-rated and child/adolescent-rated questionnaires (Total Score) were used for the present analyses.

### Participants and samples

Table 1 summarizes the demographic characteristics of the OCD subsample, the non-OCD clinical subsample, and the community sample separately for different age groups.

**Table 1:** Description of the samples

	Clinical sample (CLIN)				Community sample (COS)
	OCDS		Non-OCD		
	6–10 years old	11–18 years old	6–10 years old	11–18 years old	11–18 years old
Sample size: *N*	46	135	64	97	367
Age: Mean (SD)	9.42 (1.16)	14.42 (2.15)	9.05 (1.26)	13.80 (2.21)	14.29 (2.21)
Gender, male: *N* (%)	25 (54.3)	66 (48.9)	47 (73.4)	68 (70.1)	146 (39.8)

#### OCD subsample (OCDS)

Participants comprised 181 children and adolescents referred to the outpatient unit of the Department for Child and Adolescent Psychiatry, Psychosomatics and Psychotherapy at the Medical Faculty of the University of Cologne and the School for Child and Adolescent Cognitive Behavior Therapy at the University Hospital Cologne (n = 91, 50.30% males) and their parents. The patients’ mean age was 13.15 years (SD = 2.92; range = 6–18 years; 46 patients aged 6–10 years, 135 patients aged 11–18 years). All participants met criteria for a diagnosis of OCD (ICD diagnoses: predominantly obsessional thoughts or ruminations (F42.0): n = 15; predominantly compulsive acts, obsessional rituals (F42.1): n = 62; mixed obsessional thoughts and acts (F42.2): n = 104). The OCD diagnosis was based on a semi-structured clinical interview with the patient and the parents using the Diagnostic Checklist for OCD, which is part of the Diagnostic System for Mental Disorders in Childhood and Adolescence (DISYPS-II) [38]. Overall, 70 (38.9%) patients also had a comorbid diagnosis, consisting of tic disorders (F95, n = 19), hyperkinetic disorders (F90, n = 14), major depressive disorders (F32, n = 13), pervasive developmental disorders (F84, n = 9), emotional disorders (F93, n = 8) or phobic anxiety disorders (F40, n = 7). In total, the OCD subsample comprised 181 OCD-CA parent reports (for 46 6–10-year olds and 135 11–18-year-olds) and 134 OCD-CA self-reports.

#### Non-OCD clinical subsample (non-OCD)

This subsample comprised 161 children and adolescents referred to the same institutions described above (n = 115, 71.4% boys), with ages ranging from 6 to 18 years (M = 11.91, SD = 3.00). The most common diagnoses, primary or comorbid, were tic disorders (F95, n = 118), hyperkinetic disorders (F90, n = 30), emotional disorders (F93, n = 28), phobic anxiety disorders (F40, n = 11), reaction to severe stress and adjustment disorders (F43, n = 9), other behavioral and emotional disorders with onset usually occurring in childhood and adolescence (F98, n = 9), pervasive developmental disorders (F84, n = 7), habit and impulse disorders (F63, n = 4) and mixed disorders of conduct and emotions (F92, n = 4). In total, the non-OCD subsample comprised 161 OCD-CA parent reports (for 64 6–10-year-olds and 97 11–18-year-olds) and 84 OCD-CA self-reports.

#### Community sample (COS)

The community sample (Waclawiak 2006, unpublished) included 367 school pupils aged 11–18 years (*M *= 14.29, *SD *= 2.21; n = 146, 39.8% boys) and their caregivers (either mother or father). The participants were recruited in 11 schools in four different Federal states in Germany (North Rhine-Westphalia, Hesse, Rhineland-Palatinate, Schleswig–Holstein). 1310 OCD-CA self-report and parent-report forms were sent to the 11 schools. Questionnaires that did not meet the criteria regarding missing values < 10% were excluded. In total, 367 OCD-CA self-report forms were included in the dataset (response rate = 28%). Parent forms were only considered if they met the criteria regarding missing values and if the corresponding self-report form was present. Finally, 367 OCD-CA parent forms were selected for subsequent analysis. The CBCL and YSR were also rated by parents and pupils in the COS.

### Data analyses

To examine the factor structure of the OCD-CA in the combined OCD and non-OCD clinical sample (CLIN sample) and the OCD clinical subsample (OCDS), confirmatory factor analyses for the self-report form and the parent form were conducted separately in both samples in a first step, based on the factor structure previously found in analyses in a community sample (Waclawiak 2006, unpublished) (Additional file 1). Correlation paths between the factors were allowed because Waclawiak (2006, unpublished) found intercorrelations ≤ .65 between subscales. The tested model was assessed using x^2^ test and further fit indices. The x^2^ test examines the difference between observed and predicted data by the model, with a non-significant result indicating a good model fit. Moreover, as the x^2^ test is very sensitive to sample size, it was likely to reveal significant results considering the sizes of the assessed samples. Thus, further goodness-of-fit indices employed in comparable studies were computed to assess the model fit: the root mean square error of approximation (RMSEA), standardized root mean square (SRMR), comparative fit index (CFI) and the Tucker-Lewis index (TLI). To judge the goodness of model fit, we used the cut-off criteria proposed by Hu and Bentler [39]: RMSEA ≤ .08, better ≤ .05, SRMR ≤ .11, and CFI/TLI ≥ .80, better ≥ .95. Due to non-normally distributed data, the method of maximum-likelihood estimation was applied, using the Bollen-Stine bootstrapping (1000 samples) procedure [40].

The confirmatory factor analyses showed no satisfactory model fit (see results). Therefore, exploratory principal component analyses with varimax rotation, comprising the items of the OCD-CA, were applied in the CLIN, separately for the self-report form and the parent form. Beforehand, the data were checked with regard to their suitability for conducting exploratory principal component analyses: The Kaiser–Meyer–Olkin (KMO) and the measure of sampling adequacy (MSA) coefficient were computed, and Bartlett’s test of sphericity was carried out [40]. Additionally, as a criterion for extraction, Velicer’s (1976) minimum average partial (MAP) test and parallel analyses according to Horn were conducted to determine the number of components [40, 41].

To make the different samples comparable for further data analyses, age was divided into two groups consisting of children aged 6–10 years and adolescents aged 11–18 years (see Table 1). For analyses regarding the OCD-CA scales, raw scale scores were used. The analyses were conducted separately for the CLIN, its OCD subsample, and the COS. The non-OCD clinical subsample was only used for group comparison.

Based on the samples, descriptive analyses (means and standard deviations) for the OCD-CA subscales and the OCD Total scale were conducted. Additionally, internal consistency (Cronbach’s alphas) for the subscales developed on the basis of the principal component analyses as well as item-total correlations were calculated. For each informant (parent, child), Pearson product-moment correlations were applied for the corresponding subscales of the OCD-CA in the self-report form and the parent form. Moreover, Pearson product-moment correlations were computed to examine the relationships among the scores on the OCD-CA scales and the clinician-rated measure of OCD severity (CY-BOCS-D), the scores on parent- and self-rated measures of depressive symptoms (FBB-/SBB-DES), anxiety symptoms (FBB-/SBB-ANZ) and internalizing and externalizing problems (CBCL/YSR). ANOVAS and independent and dependent t-tests were used for group comparisons between the different samples, informants and age and gender groups regarding the OCD-CA scores (subscales and Total scale).

## Results

*Confirmatory factor* analyses in the CLIN (patients with OCD and patients with other psychological disorders) and the OCDS based on the factor structure found in the analyses of Waclawiak (2006, unpublished) did not reveal any satisfactory model fit. In none of the samples were all cut-off criteria for an acceptable model fit achieved (see Additional file 1).

Thus, *exploratory principal component analyses* with varimax rotation were conducted on the OCD-CA in the CLIN, separately for the parent form and the self-report form (Additional file 2). Data of the OCD-CA parent form consistently met criteria for conducting a factor analysis (Kaiser–Meyer–Olkin (KMO) = .90, measure of sampling adequacy coefficient: .76 ≤ MSA ≤ .96, Bartlett’s test of sphericity: x^2^ = 7077.69, df = 630, p < .001). The MAP test and parallel analysis determined four factors to be extracted. Therefore, an exploratory principal component analysis extracting four factors was applied. The four extracted factors (Additional file 2) had eigenvalues greater than 1.95 and explained 54.04% of the variance. The first factor explained 17.40% of the variance (.57 ≤ factor loadings ≤ .88) and included nine items, which describe contamination obsessions and washing compulsions (*Contamination & Washing*). The second factor explained 14.30% of the variance (.43 ≤ factor loadings ≤ .75) and consisted of 11 items describing obsessions and compulsions concerning catastrophes and injuries *(Catastrophes & Injuries)*. The third factor explained 11.39% of the variance (.36 ≤ factor loadings ≤ .73) and contained seven items describing checking compulsions *(Checking)*; item 22, describing hoarding and saving, also loads highly on this factor. The fourth factor explained 10.96% of the variance (.43 ≤ factor loadings ≤ .69) and contained five items describing ordering/arranging and repeating compulsions (*Ordering & Repeating*). Three further items regarding counting (items 20–21) and not getting ready (item 23) also load highly on the fourth factor. An additional exploratory principal component analysis with four extracted factors excluding items 20–23, which did not fit to any of the described factors in terms of content, showed the same results.

Data of the OCD-CA self-report form also met criteria for conducting a factor analysis (Kaiser–Meyer–Olkin (KMO) = .88, measure of sampling adequacy: .76 ≤ MSA ≤ .93, Bartlett’s test of sphericity: x^2^ = 3956.82, df = 630, p < .001). The MAP test suggested that five factors should be extracted. The five-factor solution did not show any meaningfully interpretable result. Parallel analysis determined four factors to be extracted. Thus, in line with the parent form, an exploratory principal component analysis extracting four factors was applied. The four-factor solution of the OCD-CA self-report form (Additional file 2) showed the following results: The four extracted factors had eigenvalues greater than 1.83 and explained 50.05% of the variance. The first factor explained 14.80% of the variance (.26 ≤ factor loadings ≤ .75) and contained six items regarding checking compulsions. A further eight items also had substantial loadings on the first factor. The second factor explained 13.67% of the variance (.54 ≤ factor loadings ≤ .78) and included nine items which describe contamination obsessions and washing compulsions. The third factor explained 10.91% of the variance (.40 ≤ factor loadings ≤ .72) and included five items describing ordering/arranging and repeating compulsions. Items 18, 20, 21, and 25, which describe compulsions regarding checking, counting and compulsions concerning catastrophes and injuries, also load (highly) on this factor. The fourth factor explained 10.67% of the variance (.45 ≤ factor loadings ≤ .74) and contained four items which describe obsessions and compulsions regarding catastrophes and injuries. Item 17 (“count and recount money”) and item 22 (“hoarding and saving”) also load highly on this factor. Although six further items describing obsessions and compulsions concerning catastrophes and injuries load on the fourth factor, all six actually load higher on other factors.

To sum up, the self-report form showed a less clear factor structure than the parent form. The factor structure of the parent form was broadly found in the self-report (see Additional file 2). For this reason, the factor structure of the parent form was used for scale formation. As items 20–23 (regarding “counting”/“certain number”, “hoarding and saving” and “not getting ready”) did not match to any of the described factors in terms of content, they were not included in any of the subscales but were included in the *Total scale*.

Exploratory principal component analyses with varimax rotation were also conducted in the OCDS, showing the same factorial solution as described for the CLIN above. Furthermore, exploratory principal axis factoring with varimax rotation also revealed no differences in the results.

To confirm the four-factor solution found in exploratory factor analyses, confirmatory factor analyses were conducted once again. Correlation paths between the factors were allowed. The x^2^ test was significant for the parent form in the CLIN (${\text{x}}^{2}_{{\left( {df = 458} \right)}}$ = 1503.170, p = .001) and OCDS (${\text{x}}^{2}_{{\left( {df = 458} \right)}}$ = 1024.023, p = .001). Further fit-indices (except for the TLI in the OCDS) indicated an acceptable factorial validity of the model (CLIN: RMSEA = .08, SRMR = .08, CFI = .83, TLI = .82; OCDS: RMSEA = .08, SRMR = .09, CFI = .80, TLI = .78).

Except for the SRMR (CLIN: .08, OCDS: .09), no fit indices met cut-off criteria for the self-report (CLIN: ${\text{x}}^{2}_{{\left( {df = 458} \right)}}$ = 1285.319, p = .001, RMSEA = .09, CFI = .74, TLI = .72; OCDS: ${\text{x}}^{2}_{{\left( {df = 458} \right)}}$ = 1013.752, p = .008, RMSEA = .09, CFI = .71, TLI = .69).

Table 2 shows the *internal consistency* (Cronbach’s alphas) and the ranges of the item-total correlations for the OCD-CA subscales and the Total scale (parent form and self-report form) across the CLIN, OCDS and COS. The Cronbach’s alpha values of the subscales and the Total scale (regarding both age groups) in the parent form were acceptable to excellent across the samples (CLIN: .78 ≤ α ≤ .94; OCDS: .74 ≤ α ≤ .93; COS: .77 ≤ α ≤ .93). The self-report form also had acceptable to excellent internal consistency, with the exception of the subscale *Ordering & Repeating* in the COS (CLIN: .74 ≤ α ≤ .93; OCDS: .70 ≤ α ≤ .92; COS: .55 ≤ α ≤ .91). Item-total correlations were generally satisfactory. Although several items had low item-total correlations (rit < .30), excluding any of these items did not noticeably change the Cronbach’s alpha.

**Table 2:** OCD-CA parent form and self-report form: Cronbach’s alphas (α) and item-total correlations, CLIN, {OCDS}, (COS)

Scale	Parent form				Self-report form	
	6–10 years old		11–18 years old		11–18 years old	
	α	Item-total r	α	Item-total r	α	Item-total r
Contamination & Washing (9 items)	.91 {.91}	.55–.83 {.49-.83}	.94 {.93} (.85)	.62–.89 {.54–.87} (.47–.69)	.89 {.88} (.78)	.55–.71 {.54–.72} (.31–.60)
Catastrophes & Injuries (11 items)	.88 {.88}	.42–.76 {.35-.76}	.87 {.85} (.84)	.25–.74 {.16–.73} (.28–.73)	.87 {.87} (.82)	.43–.71 {.41–73} (.36–.64)
Checking (7 items)	.80 {.83}	.33–.67 {40-.69}	.82 {.81} (.80)	.43-.68 {.37–.68} (.31–.64)	.78 {.79} (.74)	.41–.62 {.40–.64} (.34–.55)
Ordering & Repeating (5 items)	.78 {.74}	.49–.67 {.33-.63}	.84 {.80} (.77)	.60–.75 {.53–.69} (.48–.63)	.74 {.70} (.55)	.49–.54 {.43–.53} (.11–.49)
OCD Total (36 items)	.92 {.90}	.18–.69 {.18-.71}	.93 {.88} (.93)	.18-.67 {.08-.54} (.23–.66)	.93 {.92} (.91)	.35–.68 {.29–.66} (.14–.61)

Parent-report form: 6–10 years old: n = 110, {n = 46}; 11–18 years old: n = 232, {n = 134}, (n = 367)

Self-report form: n = 218, {n = 134}, (n = 367)

The *intercorrelations* of the subscales in the parent form (Additional file 3) yielded different results across the samples. In the CLIN, the subscales showed low to high intercorrelations (.23 ≤ r ≤ .61). All intercorrelations were significant at a level of .01 (except for the intercorrelation between the subscale *Contamination & Washing* and the subscale *Checking*, p < .05). In the OCDS, low and moderate intercorrelations emerged (.05 ≤ r ≤ .51, partially significant at a level of p < .01 or p < .05). High intercorrelations were found in the COS (.55 ≤ r ≤ .71, p < .01). The intercorrelations of the subscales in the self-report form (Additional file 4) yielded similar, comparable results across the samples. Subscales showed moderate to high significant intercorrelations (.32 <= r <=.71, p < .01), with the exception of the subscales *Contamination & Washing* and *Ordering & Repeating* in the OCDS (r = .28, p < .01, low and significant correlation).

The correlations between the corresponding OCD-CA subscales and Total scores of the parent form and self-report form (Table 3) were generally moderate to high and significant (.32 ≤ r ≤ .68, p < .01), with the exception of the correlations of the corresponding subscales *Contamination & Washing* (r = .27, p < .01) and *Catastrophes & Injuries* (r = .29, p < .01) in the COS, which were significant but low.

**Table 3:** Correlation between corresponding scales in the parent and self-report form, CLIN, {OCDS}, (COS)

Scale	r parent-/self-report
Contamination & Compulsions	.68 {.65} (.27)
Catastrophes & Injuries	.47 {.44} (.29)
Checking	.55 {.54} (.32)
Ordering & Repeating	.53 {.43} (.46)
OCD Total	.54 {.44} (.32)

All correlations significant at p < .01; n = 218, {n = 134}, (n = 367)

### Convergent and divergent validity

Correlations between the OCD-CA scales of the parent form and self-report form, respectively, and other scales assessing anxiety, depression, and internalizing and externalizing problems in the CLIN (divided into two age groups) are reported in Table 4. Predominantly moderate correlations were found between the parent-rated/self-rated *OCD*-*CA Total* scores on the one hand and parent-rated/self-rated *Internalizing Problems, Anxiety Symptoms* and *Depression Symptoms* on the other, while correlations with *Externalizing Problems* were lower. The correlations of the OCD-CA subscales with other ratings were predominantly close to those of the OCD-CA Total scores, with the exception of the subscale *Checking*, which had mainly lower correlations. Correlations in the other samples (OCDS, COS) were similar (Additional file 5, 6).

**Table 4:** CLIN: Correlations between the OCD-CA scales and internalizing and externalizing problems and symptoms

OCD-CA scales	CBCL/YSR		FBB-/SBB-DES Total score	FBB-/SBB-ANZ Total score
	Internalizing problems	Externalizing problems		
Contamination & Washing	.54** [.32**] (.30**)	.02 [.17**] (.22**)	.49** [.22**] (.25**)	.54** [.39**] (.29**)
Catastrophes & Injuries	.64** [.46**] (.54**)	.02 [.24**] (.33**)	.56** [.30**] (.48**)	.63** [.67**] (.66**)
Checking	.19 [.30**] (.45**)	.04 [.16*] (.28**)	.18 [.21**] (.38**)	.24* [.50**] (.50**)
Ordering & Repeating	.33** [.34**] (.34**)	− .01 [.26**] (.19**)	.39** [.31**] (.32**)	.39** [.37**] (.35**)
OCD Total	.59** [.49**] (.52**)	.03 [.29**] (.34**)	.58** [.38**] (.46**)	.62** [.67**] (.57**)

Parent form/(self-report form); CLIN: 6–10 years old and [11–18 years old]

* p < .05, ** p < .01; CBCL: n = 105, FBB-DES: n = 92, FBB-ANZ: n = 69, [CBCL: n = 224, FBB-DES: n = 203, FBB-ANZ: n = 164]; (YSR: n = 210, SBB-DES: n = 199, SBB-ANZ: n = 162)

Correlations between the *self*-*rated OCD*-*CA Total score* and the clinician-rated *CY*-*BOCS*-*D Total score* were in the moderate range (r = .53) and higher than the correlations between *parent*-*rated OCD*-*CA scale scores* and the *CY*-*BOCS*-*D Total score*, which were not statistically significant (Additional file 7). The parent-rated OCD-CA scales correlated with the content-corresponding subscales of the CY-BOCS-D Checklist. These correlations were statistically significant (p < .05) in the small to moderate range (.23 ≤ r ≤ .69), with the exception of the correlation between the OCD-CA subscale *Catastrophes & Injuries* and the CY-BOCS-D Checklist subscale Repeating, ordering/arranging, hoarding and magical thinking (r = .12). No significant correlations were found on the non-corresponding subscales. The self-rated OCD-CA scale scores also correlated statistically significantly (p < .01) in the low to high range (.30 ≤ r ≤ .75) with the content-corresponding subscales of the CY-BOCS-D Checklist. Only two significant correlations were found for the non-corresponding subscales (Additional file 7).

### Comparisons of means between samples and informants, age and gender effects

Table 5 presents the mean scores and standard deviations of the OCD-CA subscales and Total scale for the OCDS, non-OCD and COS for the age group 11–18 years. ANOVAs (one-way) revealed significant (p < .001) group differences on the OCD-CA Total and subscale scores between these groups. Post hoc comparisons showed that the OCDS scored significantly higher than the non-OCD and the COS on all scales in the parent form and the self-report form. Additionally, in the self-report form, the COS scored significantly higher (p < .05) than the non-OCD on the scale *Contamination & Washing* and the OCD *Total Score*.

**Table 5:** Comparison of means between clinical OCDS and Non-OCD and COS (11–18-year-olds) (ANOVA)

Scale	Sample	Parent form			Self-report form		
		N	M (SD)	F	N	M (SD)	F
Contamination & Washing	OCDS	135	13.06 (10.91)^a^	128.32**	134	9.96 (8.39)^a^	36.23**
	Non-OCD	97	3.02 (5.59)^b^		84	3.81 (5.61)^bc^	
	COS	367	2.89 (4.05)^b^		367	5.54 (4.77)^bd^	
Catastrophes & Injuries	OCDS	135	9.28 (8.53)^a^	95.07**	134	9.72 (9.19)^a^	25.99**
	Non-OCD	97	2.80 (5.36)^b^		84	4.07 (4.87)^b^	
	COS	367	1.94 (3.50)^b^		367	5.49 (5.65)^b^	
Checking	OCDS	135	4.36 (5.08)^a^	44.60**	134	5.54 (5.43)^a^	12.88**
	Non-OCD	97	1.09 (2.31)^b^		84	2.55 (3.02)^b^	
	COS	367	1.43 (2.54)^b^		367	4.59 (4.03)^b^	
Ordering & Repeating	OCDS	135	6.10 (5.32)^a^	172.65**	134	5.56 (4.50)^a^	102.99**
	Non-OCD	97	0.95 (2.12)^b^		84	1.46 (2.54)^b^	
	COS	367	0.65 (1.67)^b^		367	1.51 (2.08)^b^	
OCD Total	OCDS	135	36.30 (20.70)^a^	198.11**	134	34.31 (23.26)^a^	49.38**
	Non-OCD	97	9.38 (14.69)^b^		84	13.51 (14.53)^bc^	
	COS	367	8.16 (11.01)^b^		367	19.39 (14.83)^bd^	

** p < .001

^a,b^Samples differ significantly at a level of < .001; ^c,d^ samples differ significantly at a level of < .05

Within the clinical sample of 6–10-year-old children, parent-rated OCD-CA scores were higher in the OCD subsample than in the non-OCD subsample (Additional file 8).

In the OCD subsample, no significant differences were found between the self-rated and the parent-rated OCD-CA total scores, while in the COS, self-reported OCD-CA total scores and subscale scores were higher than parent-reported scores. Within the OCD sample, higher parent ratings were found for *Contamination & Washing* and lower parent ratings emerged for *Checking* (Additional file 9).

Significant age effects were found within the CLIN (parent form) across all scales except for the scale *Ordering and Repeating*. Parents of 11–18-year-olds gave higher ratings than parents of 6–10-year-olds. Gender effects only emerged on the scale *Checking*. Parents of girls provided significantly higher ratings than parents of boys on this scale (Additional file 10). Within the OCD subsample, no age or gender effects were found on the OCD-CA subscales and the Total score, with the exception of the subscale *Contamination & Washing* (Additional file 11).

Within the CLIN (self-report form), significantly higher ratings for girls than for boys were found on the scales *Catastrophes & Injuries*, *Ordering & Repeating* and the *OCD Total scale*. No significant mean gender differences were found in the COS, with the exception of the subscale *Ordering & Repeating* in the parent form (Additional file 12).

## Discussion

The aim of this study was to examine the psychometric properties of a new parent-rated and self-rated inventory for pediatric obsessive-compulsive disorder, the OCD-CA, across a clinical sample comprising an OCD subsample and a non-OCD clinical subsample, as well as a community sample. For the total clinical sample and the OCD subsample, confirmatory factor analyses were unable to replicate the factor structure found in a community sample in a previous study (Waclawiak 2006, unpublished). Thus, exploratory principal component analysis with varimax rotation was conducted, resulting in a four factor-solution: (1) *Contamination & Washing*, *(2) Catastrophes & Injuries*, *(3) Checking*, and *(4) Ordering & Repeating*. Internal consistency was acceptable to excellent for all subscales (except for the self-report subscale *Ordering & Repeating* in the COS) and for the *Total scale* across the samples (CLIN, OCDS, COS). Therefore, internal consistency is comparable to that of other OCD-specific assessment instruments examined in OCD patients (e.g. Scahill et al. [21]; Storch et al. [14]). In contrast to the CY-BOCS-CR [17], but in line with the OCI-CV [7, 42–44], good internal consistency was also confirmed in a community sample.

Intercorrelations between the subscales mainly lay at r ≤ .70, with the exception of those between the subscales *Catastrophes & Injuries* and *Checking* (r = .71) and *Checking* and *Ordering and Repeating* (r = .71) in the COS (parent form: 11–18 years old), and between *Catastrophes & Injuries* and *Checking* (r = .71) in the CLIN (self-report). The intercorrelations of the self-report subscales in the OCD subsample were similar to or higher than those found in analyses of the OCI-CV [42].

Thus, subscales of the OCD-CA are generally sufficiently independent of each other [45].

The correlations between the corresponding OCD-CA subscales and Total scale of the parent form and self-report form were generally moderate to high and statistically significant, which is in line with results reported by Shafran et al. [15], Uher et al. [16], and Storch et al. [8].

In the OCD subsample, self-rated and parent-rated corresponding scales only demonstrated significant mean differences on two scales with opposite tendencies, while Storch et al. [8] demonstrated significantly lower self-rated scores than parent-rated scores in an OCD sample. However, significant mean differences between informants were found across all scales in the COS, with children/adolescents providing higher scores than their parents. It might be assumed that children/adolescents from a mainly healthy population have not discussed the assessed OCD symptoms with their parents, while those affected by OCD (and who have already visited outpatient departments) are likely to have communicated with their parents about their obsessions and compulsions. This finding might also indicate that some of the symptoms of OCD (e.g. obsessions) might be more difficult for other people to detect [12].

With regard to convergent validity, the self-reported OCD-CA Total score correlated moderately with the clinician-rated CY-BOCS-D Total Score in the OCD sample. In other studies, moderate to large correlations between pediatric OCD assessments and the CY-BOCS were only found when the assessed instruments also focused on more global severity assessment, unrelated to the number and type of symptoms (e.g. CHOCI Impairment Scale [15]). Instruments assessing OCD symptoms in different domains usually found lower correlations with the CY-BOCS Rating Scale Total Score [7, 42, 46]. In contrast, parent ratings on the OCD-CA did not correlate with the CY-BOCS-D Total Score. This difference between parent ratings and self-reports on the OCD-CA may be due to the fact that the clinicians rated the CY-BOCS-D primarily based on an interview with the child or adolescent.

The correlations between the OCD-CA scales and the corresponding CY-BOCS-D Checklist scales (also focusing on OCD symptom dimensions) were higher than correlations with the Total scale of the CY-BOCS-D Rating Scale.

Correlations between the OCD-CA Total scores (parent- and self-reported) and measures of internalizing problems, depressive symptoms and anxiety symptoms were predominantly moderate to high across samples, which is in line with other studies [7, 8, 46].

To sum up, correlations between the OCD-CA and the CY-BOCS-D as well as measures of internalizing problems, depressive symptoms and anxiety symptoms provided support for convergent validity.

Discriminant validity of the OCD-CA was confirmed by (negative) low to moderate correlations between the self-report/parent form and the subscale Externalizing Problems of the CBCL and YSR. Other studies found exclusively low correlations between pediatric OCD measures and the subscale Externalizing Problems of the CBCL (e.g. Storch et al. [8]).

Regarding discriminant validity, in line with expectation, the OCD-CA scores in the OCD subsample were significantly higher than those in the non-OCD subsample and the COS sample.

The strengths of the current study include the evaluation of a new pediatric OCD-specific assessment, including a self-report and a parent-report form, across three samples (CLIN, OCDS, COS) with large sample sizes. However, some limitations should also be mentioned: First, with regard to the samples, the COS was not a representative sample, and the CLIN consisted mainly of patients with tic disorders and OCD as the data were collected at the corresponding outpatient departments of the described institutions. Second, the exploratory factor analysis did not show an adequate fit for any clearly interpretable model for the self-rated OCD-CA. Furthermore, except for the SRMR, the values resulting from the confirmatory factor analysis did not indicate goodness of fit of the model. Accordingly, the factorial validity of the self-report form could not be confirmed. Nevertheless, based on the parent report model, reliability and validity of the self-report form were confirmed. Overall, internal consistency, factorial validity (for the parent version only), and convergent und divergent validity of the new rating scale were confirmed. However, the OCD-CA should be examined further by other research teams based on the EBA criteria.

## Conclusion

Due to the lack of instruments assessing self-rated and parent-rated symptoms across common OCD domains, this study aimed to evaluate a German version of the Padua Inventory-Washington State University Revision which enables to measure pediatric OCD and records both self- and parent report regarding OCD symptom domains. Accordingly, the OCD-CA supports multiple-informant assessment to achieve a comprehensive clinical picture of the disorder. Overall, the results of the present study show that the OCD-CA is a promising, valid and reliable instrument to assess self-rated and parent-rated pediatric OCD symptoms in clinical and non-clinical (community) populations.

## Additional files

**Additional file 1.** Results from confirmatory factor analyses based on the four-factor solution by Waclawiak (2006; unpublished). The four-factor solution found by Waclawiak (2006; unpublished) is illustrated, and results from confirmatory factor analyses based on this four-factor solution and conducted in the CLIN and OCDS are summarized.

**Additional file 2.** Exploratory principal component analysis with varimax rotation, four-factor solution. Results of the four-factor solution of the OCD-CA parent- and self-report form are shown.

**Additional file 3.** Parent form: Intercorrelations between the subscales. Intercorrelations between the OCD-CA subscales in the parent form across the OCD subsample (OCDS), the combined clinical sample (CLIN) and the community sample (COS) are shown.

**Additional file 4.** Self-report form: Intercorrelations between the subscales. Intercorrelations between the OCD-CA subscales in the self-report form across the OCD subsample (OCDS), the combined clinical sample (CLIN) and the community sample (COS) are shown.

**Additional file 5.** OCDS: Correlations between the OCD-CA scales and internalizing and externalizing problems and symptoms. Correlations between the OCD-CA scales of the parent form and self-report form, respectively, and other scales assessing anxiety, depression, and internalizing and externalizing problems in the OCD subsample (divided into two age groups) are reported.

**Additional file 6.** COS: Correlations between the OCD-CA scales and internalizing and externalizing problems. Correlations between the OCD-CA scales of the parent form and self-report form, respectively, and other scales assessing internalizing and externalizing problems in the community subsample are reported.

**Additional file 7.** OCDS: Correlations between the OCD-CA scales of the parent form/(self-report form) and the CY-BOCS-D. Correlations between the self-rated OCD-CA/parent-rated OCD-CA and the clinician-rated CY-BOCS-D in the OCD subsample of the 11 to 18 years old are reported.

**Additional file 8.** Comparison of OCD-CA parent ratings in the OCDS and non-OCD in children aged 6 to 10 years old. OCD-CA parent ratings of the 6 to 10 years old children in the OCD subsample and the non-OCD clinical subsample (patients with other psychological disorders) are compared.

**Additional file 9.** Comparison of means between self- and parent-report form. In the OCD subsample and the community sample self-rated and parent-rated OCD-CA mean scale scores are compared.

**Additional file 10.** CLIN: Comparison of means between age groups and gender in the parent form (ANOVA). Results of ANOVA in the combined clinical sample regarding comparison of means between age groups (6–10 years old and 11–18 years old) and gender in the parent form are presented.

**Additional file 11.** OCDS: Comparison of means between age groups and gender in the parent form (ANOVA). Results of ANOVA in the OCD subsample regarding comparison of means between age groups (6–10 years old and 11–18 years old) and gender in the parent form are presented.

**Additional file 12.** Comparison of means between boys and girls. Results of ANOVA in the combined clinical sample, OCD subsample and community sample regarding comparison of means between gender in the parent and self-report form are reported.

## Data Availability

The datasets used and/or analyzed during the current study are available from the corresponding author on reasonable request.

## Abbreviations

OCD-CA: OCD Inventory for Children and Adolescents
OCD: obsessive-compulsive disorder
CY-BOCS: Children’s Yale-Brown Obsessive-Compulsive Scale
CY-BOCS-CR: Child-report version of the Children’s Yale-Brown Obsessive-Compulsive Scale
CY-BOCS-PR: Parent-report version of the Children’s Yale-Brown Obsessive-Compulsive Scale
CHOCI: Children’s Obsessional Compulsive Inventory
CHOCI-R: Children’s Obsessional Compulsive Inventory-Revised
SBB-ZWA: Self-rated German Symptom Checklist for Obsessive-Compulsive and Related Disorders
FBB-ZWA: Parent-rated German Symptom Checklist for Obsessive-Compulsive and Related Disorders
EBA: evidence-based assessment
OCI-CV: Obsessive Compulsive Inventory-Child Version
ZWIK: Zwangsinventar für Kinder und Jugendliche
PI-WSUR: Padua Inventory-Washington State University Revision
PI: Padua Inventory
CY-BOCS-D: German version of the Children’s Yale-Brown Obsessive-Compulsive Scale
CBCL/6-18R: German version of the Child Behavior Checklist
YSR/11-18R: German version of the Youth Self Report
FBB-ANZ: Parent-rated German Symptom Checklist for Anxiety and Obsessive-Compulsive Disorders
SBB-ANZ: Self-rated German Symptom Checklist for Anxiety and Obsessive-Compulsive Disorders
ICD-10: tenth edition of the International Statistical Classification of Diseases and Related Health Problems
DSM-IV: fourth edition of the Diagnostic and Statistical Manual of Mental Disorders
DISYPS-II: Diagnostic System for the Assessment of Mental Disorders in Children and Adolescents based on the ICD-10 and DSM-IV
FBB-DES: Parent-rated German Symptom Checklist for Depressive Disorders
SBB-DES: Self-rated German Symptom Checklist for Depressive Disorders
OCDS: clinical subsample including patients diagnosed with obsessive-compulsive disorders
Non-OCD: clinical subsample including patients diagnosed with other psychological disorders than obsessive-compulsive disorders
COS: community sample
CLIN: combined sample including patients with obsessive-compulsive disorders and other psychological disorders
RMSEA: root mean square error of approximation
SRMR: standardized root mean square
CFI: comparative fit index
TLI: Tucker-Lewis index
KMO: Kaiser-Meyer-Olkin
MSA: measure of sampling adequacy
MAP: Velicer’s minimum average partial
