# Supplementary material for: Psychometric evaluation of a parent-rating and self-rating inventory for pediatric obsessive-compulsive disorder: German OCD Inventory for Children and Adolescents (OCD-CA)
Source: Child Adolesc Psychiatry Ment Health. 2019 Jun 18;13:25. doi: 10.1186/s13034-019-0286-z (PMC6582526; doi:10.1186/s13034-019-0286-z)
Supplement: Supplementary file 1 — Additional file 1. Results from confirmatory factor analyses based on the four-factor solution by Waclawiak (2006; unpublished). The four-factor solution found by Waclawiak (2006; unpublished) is illustrated, and results from confirmatory factor analyses based on this four-factor solution and conducted in the CLIN and OCDS are summarized. [file 13034_2019_286_MOESM1_ESM.pdf]

Additional File 1

Results from confirmatory factor analyses based on the four-factor solution by Waclawiak (2006; unpublished)

The tested model/ four-factor solution found by Waclawiak (2006, unpublished)

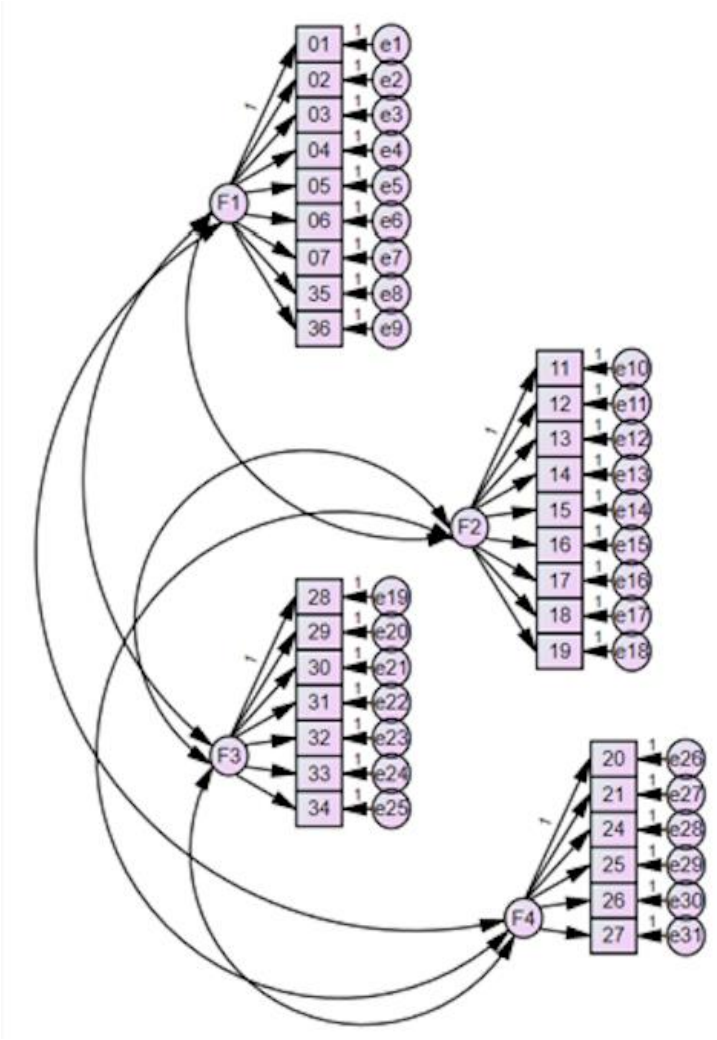

**F1**=Factor 1	**F2**=Factor 2	**F3**=Factor 3	**F4**=Factor 4
Item **01**: Hands feel dirty	Item **11**: Doing things several times	Item **28**: Unnecessary concerns	Item **20**: Certain number
Item **02**: Difficulties to touch certain objects	Item **12**: Repetitive checking more often than necessary	Item **29**: Fear of something bad happening	Item **21**: Counting
Item **03**: Difficulties to touch garbage	Item **13**: Checking and rechecking water taps or light switches	Item **30**: Worrying about having hurt someone	Item **24**: Certain actions to avoid misfortune
Item **04**: Avoiding using public toilets	Item **14**: Checking doors, windows or drawers	Item **31**: Fear of being to blame for catastrophes	Item **25**: Certain numbers or words to keep away bad luck
Item **05**: Intensive hand-washing	Item **15**: Checking homework	Item **32**: Worrying about having a disease	Item **26**: Recurrent thoughts
Item **06**: Hand-washing due to fear of contamination	Item **16**: Checking matches, candles etc.	Item **33**: Getting worried at the sight of pointed objects	Item **27**: Reassurance-seeking
Item **07**: Washing due to thoughts of being dirty	Item **17**: Counting and recounting money	Item **34**: Getting upset when hearing about a crime	
Item **35**: Useless worries about germs or toxins	Item **18**: Rereading texts several times		
Item **36**: Disgust for perspiration or urine	Item **19**: Repeating actions		

Note: The model was tested using AMOS [Arbuckle JL. Amos (Version 23.0) [Computer Program]. IBM SPSS: Chicago; 2014.]

Results from confirmatory factor analyses based on the four-factor solution by Waclawiak (2006, unpublished)

**Sample**	**x^2 test**	**RMSEA**	**90%Cl**	**SRMR**	**CFI**	**TLI**
CLIN	$x^2_{(df=428)}$= 1553.380, p=.001	.09	.08-.09	**.08**	**.81**	**.80**
	($x^2_{(df=428)}$= 1205.661, p=.001)	(.09)	(.09-.10)	(**.08**)	(.75)	(.73)
OCDS	$x^2_{(df=428)}$= 1036.113, p=.001	.09	.08-.10	**.09**	.78	.76
	($x^2_{(df=428)}$= 1005.776, p=.004)	(.10)	(.09-.11)	(**.09**)	(.70)	(.67)

Note: CLIN: parent form: n=342, (self-report form: n=218); OCDS: parent form: n=181, (self-report form=134); values printed in bold met cut-off criteria for goodness of model fit.
