# Supplementary material for: Psychometric evaluation of a parent-rating and self-rating inventory for pediatric obsessive-compulsive disorder: German OCD Inventory for Children and Adolescents (OCD-CA)
Source: Child Adolesc Psychiatry Ment Health. 2019 Jun 18;13:25. doi: 10.1186/s13034-019-0286-z (PMC6582526; doi:10.1186/s13034-019-0286-z)
Supplement: Supplementary file 2 — Additional file 2. Exploratory principal component analysis with varimax rotation, four-factor solution. Results of the four-factor solution of the OCD-CA parent- and self-report form are shown. [file 13034_2019_286_MOESM2_ESM.pdf]

Additional file 2

Exploratory principal component analysis with varimax rotation, four-factor solution

	Parent form: Factors				Self-report form: Factors			
**Item**	**1**	***2***	**3**	**4**	**1**	**2**	**3**	**4**
Contamination & Washing								
1. Hands feel dirty	**.86**					**.78**		
2. Difficulties to touch certain objects	**.82**					**.73**		
3. Difficulties to touch garbage	**.82**					**.75**		
4. Avoiding using public toilets	**.76**					**.65**		
5. Intensive hand-washing	**.83**					**.73**	.32	
6. Hand-washing due to fear of contamination	**.85**					**.71**	.28	
7. Washing due to thoughts of being dirty	**.88**					**.71**		
35. Useless worries about germs or toxins	**.57**				.40	**.54**		
36. Disgust for perspiration or urine	**.72**	.50				**.61**		
Catastrophes & Injuries								
24. Certain actions to avoid misfortune		**.66**		.39	.51		.44	**.29**
25. Certain numbers or words to keep away bad luck		**.56**		.33	.31		.55	**.34**
26. Recurrent thoughts		**.62**	.25		.53			**.45**
27. Reassurance-seeking		**.67**	.27		**.67**			
28. Unnecessary concerns		**.75**	.29		.58			**.42**
29. Fear of something bad happening		**.66**	.41		.65			**.30**
30. Worrying about having hurt someone		**.50**	.33		.44			**.45**
31. Fear of being to blame for catastrophes		**.61**						**.63**
32. Worrying about having a disease		**.67**			.51			**.37**
33. Getting worried at the sight of pointed objects		**.43**						**.64**
34. Getting upset when hearing about a crime		**.69**						**.74**
Checking								
12. Repetitive checking more often than necessary			**.56**	.44	**.75**			
13. Checking and rechecking water taps or light switches			**.67**		**.72**			
14. Checking doors, windows or drawers			**.73**		**.65**			
15. Checking homework			**.70**	.25	**.39**			
16. Checking matches, candles etc.		.26	**.65**		**.49**			
17. Counting and recounting money			**.59**		**.26**		.25	.53
18. Rereading texts several times			**.58**				**.53**	.43
22. Hoarding and saving			.36					.64
Ordering & Repeating								
8. Following a particular order in washing	.40			**.65**		.32	**.63**	
9. Doing certain things in a certain order before going to sleep				**.61**			**.70**	
10. Hanging up or folding clothes in a special way				**.65**			**.72**	
11. Doing things several times			.42	**.69**	.57		**.40**	
19. Repeating actions		.31	.30	**.65**	.41		**.53**	
20. Certain number		.28		.67			.60	.33
21. Counting				.55			.53	.32
23. Not getting ready	.27			.43	.26			

Note: CLIN: parent form: n=342, self-report form: n=218; abbreviated item content; factor loadings > .25
