# Supplementary material for: Psychometric evaluation of a parent-rating and self-rating inventory for pediatric obsessive-compulsive disorder: German OCD Inventory for Children and Adolescents (OCD-CA)
Source: Child Adolesc Psychiatry Ment Health. 2019 Jun 18;13:25. doi: 10.1186/s13034-019-0286-z (PMC6582526; doi:10.1186/s13034-019-0286-z)
Supplement: Supplementary file 3 — Additional file 3. Parent form: Intercorrelations between the subscales. Intercorrelations between the OCD-CA subscales in the parent form across the OCD subsample (OCDS), the combined clinical sample (CLIN) and the community sample (COS) are shown. [file 13034_2019_286_MOESM3_ESM.pdf]

Additional file 3
Parent form: Intercorrelations between the subscales

	Parent form					
**Scale**	**CAT**		**CHECK**		**Ordering & Repeating**	
	**6-10 years old**	**11-18 years old**	**6-10 years old**	**11-18 years old**	**6-10 years old**	**11-18 years old**
**Contamination & Washing (CONT)**	.56** {.47**}	.36** {.11} (.55**)	.23* {.05}	.29** {.06} (.65**)	.46** {.33*}	.35** {.08} (.60**)
**Catastrophes & Injuries (CAT)**			.31** {.15}	.54** {.41**} (.71**)	.40** {.25}	.45** {.27**} (.69**)
**Checking (CHECK)**					.61** {.51**}	.55** {.43**} (.71**)

Note: CLIN, {OCDS}, (COS); 6-10 years old: n=110, {n=46}; 11-18 years old: n=232, {n=134}, (n=367);
*p<.05, **p<.01
