# Supplementary material for: Psychometric evaluation of a parent-rating and self-rating inventory for pediatric obsessive-compulsive disorder: German OCD Inventory for Children and Adolescents (OCD-CA)
Source: Child Adolesc Psychiatry Ment Health. 2019 Jun 18;13:25. doi: 10.1186/s13034-019-0286-z (PMC6582526; doi:10.1186/s13034-019-0286-z)
Supplement: Supplementary file 4 — Additional file 4. Self-report form: Intercorrelations between the subscales. Intercorrelations between the OCD-CA subscales in the self-report form across the OCD subsample (OCDS), the combined clinical sample (CLIN) and the community sample (COS) are shown. [file 13034_2019_286_MOESM4_ESM.pdf]

Additional file 4
Self-report form: Intercorrelations between the subscales

Self-report form, 11-18 years old			
**Scale**	**CAT**	**CHECK**	**Ordering & Repeating**
**Contamination & Washing (CONT)**	.43**<br>{.32**}<br>(.53**)	.46**<br>{.32**}<br>(.55**)	.44**<br>{.28**}<br>(.54**)
**Catastrophes & Injuries (CAT)**		.71**<br>{.68**}<br>(.63**)	.59**<br>{.53**}<br>(.55**)
**Checking (CHECK)**			.61**<br>{54**}<br>(.62**)

Note: CLIN: n=218, {OCDS: n=134}, (COS: n=367);
**p<.01
