# Supplementary material for: Psychometric evaluation of a parent-rating and self-rating inventory for pediatric obsessive-compulsive disorder: German OCD Inventory for Children and Adolescents (OCD-CA)
Source: Child Adolesc Psychiatry Ment Health. 2019 Jun 18;13:25. doi: 10.1186/s13034-019-0286-z (PMC6582526; doi:10.1186/s13034-019-0286-z)
Supplement: Supplementary file 5 — Additional file 5. OCDS: Correlations between the OCD-CA scales and internalizing and externalizing problems and symptoms. Correlations between the OCD-CA scales of the parent form and self-report form, respectively, and other scales assessing anxiety, depression, and internalizing and externalizing problems in the OCD subsample (divided into two age groups) are reported. [file 13034_2019_286_MOESM5_ESM.pdf]

Additional file 5

OCDS: Correlations between the OCD-CA scales and internalizing and externalizing problems and symptoms

OCD-CA Scales	CBCL/ YSR		FBB-/SBB-DES Total Score	FBB-/ SBB-ANZ Total Score
	Internalizing Problems	Externalizing Problems		
Contamination & Washing	.55** [.23**] (.23**)	-.10 [.16] (.20*)	.45** [.15] (.18*)	.53** [.21*] (.28*)
Catastrophes & Injuries	.69** [.43**] (.55**)	-.09 [.22*] (.39**)	.49** [.26**] (.46**)	.63** [.63**] (.68**)
Checking	.10 [.23**] (.47**)	-.04 [.14] (.32**)	.06 [.16] (.34**)	.11 [.46**] (.52**)
Ordering & Repeating	.23 [.30**] (.33**)	-.17 [.33**] (.31**)	.28 [.32**] (.33**)	.25 [.27**] (.38**)
OCD Total	.61** [.48**] (.54**)	-.13 [.34**] (.43**)	.52** [.37**] (.45**)	.60** [.62**] (.61**)

Note: parent form: 6-10 years old and [11-18 years old] / (self-report form); parent form: CBCL: n=42, FBB-DES: n=40, FBB-ANZ: n=35, [CBCL: n=132, FBB-DES: n=127, FBB-ANZ: n=101]; self-report form: (YSR: n=130, SBB-DES: n=126, SBB-ANZ: n=101);

*p<.05, **p<.01
