# Supplementary material for: Psychometric evaluation of a parent-rating and self-rating inventory for pediatric obsessive-compulsive disorder: German OCD Inventory for Children and Adolescents (OCD-CA)
Source: Child Adolesc Psychiatry Ment Health. 2019 Jun 18;13:25. doi: 10.1186/s13034-019-0286-z (PMC6582526; doi:10.1186/s13034-019-0286-z)
Supplement: Supplementary file 6 — Additional file 6. COS: Correlations between the OCD-CA scales and internalizing and externalizing problems. Correlations between the OCD-CA scales of the parent form and self-report form, respectively, and other scales assessing internalizing and externalizing problems in the community subsample are reported. [file 13034_2019_286_MOESM6_ESM.pdf]

Additional file 6
COS: Correlations between the OCD-CA scales and internalizing and externalizing problems

OCD-CA scales	CBCL/ YSR	
	**Internalizing Problems**	**Externalizing Problems**
Contamination & Washing	.39**	.27**
	(.23**)	(.22**)
Catastrophes & Injuries	.65**	.47**
	(.43**)	(.37**)
Checking	.44**	.23**
	(.28**)	(.22**)
Ordering & Repeating	.45**	.24**
	(.22**)	(.19**)
OCD Total	.60**	.40**
	(.39**)	(.34**)

Note: parent form/ (self-report form); parent form: CBCL: n=246-257; self-report form: (YSR: n=354-357); *p<.05, **p<.01;
