# Supplementary material for: Psychometric evaluation of a parent-rating and self-rating inventory for pediatric obsessive-compulsive disorder: German OCD Inventory for Children and Adolescents (OCD-CA)
Source: Child Adolesc Psychiatry Ment Health. 2019 Jun 18;13:25. doi: 10.1186/s13034-019-0286-z (PMC6582526; doi:10.1186/s13034-019-0286-z)
Supplement: Supplementary file 7 — Additional file 7. OCDS: Correlations between the OCD-CA scales of the parent form/(self-report form) and the CY-BOCS-D. Correlations between the self-rated OCD-CA/parent-rated OCD-CA and the clinician-rated CY-BOCS-D in the OCD subsample of the 11 to 18 years old are reported. [file 13034_2019_286_MOESM7_ESM.pdf]

Additional file 7

OCDS: Correlations between the OCD-CA scales of the parent form/ (self-report form) and the CY-BOCS-D

	**CY-BOCS-D Rating Scale**	**CY-BOCS-D Checklist Scales**				
**OCD-CA scales**	OCD total Severity	Obsessions regarding loss of control and religion	Checking, harm avoidance and sexual obsessions	Contamination and Cleaning	Repeating, ordering/arranging, hoarding and magical thinking	OCD Total
Contamination & Washing	.10	-.13	-.07	**.69****	-.15	.13
	(.27)	(-.01)	(.03)	**(.75**)**	(.01)	(.32**)
Catastrophes & Injuries	.19	**.32****	**.38****	-.08	**.12**	.28**
	(.41**)	**(.30**)**	**(.60**)**	(-.09)	**(.39**)**	(.49**)
Checking	.27	.02	**.43****	-.01	.20	.27**
	(.38*)	(.15)	**(.54**)**	(-.02)	(.37**)	(.44**)
Ordering & Repeating	-.03	-.13	.07	.10	**.23***	.12
	(.51**)	(.17)	(.28**)	(.11)	**(.48**)**	(.44**)
OCD-CA Total	.21	.04	.26*	.36**	.13	**.32****
	(.53**)	(.23*)	(.49**)	(.27**)	(.44**)	**(.60**)**

Note: OCDS: 11-18 years old; CY-BOCS-D Rating Scale: n=44, (n=44); CY-BOCS-D Checklist Scales: n=90, (n=92); *p<.05, **p<=.01
