# Supplementary material for: Psychometric evaluation of a parent-rating and self-rating inventory for pediatric obsessive-compulsive disorder: German OCD Inventory for Children and Adolescents (OCD-CA)
Source: Child Adolesc Psychiatry Ment Health. 2019 Jun 18;13:25. doi: 10.1186/s13034-019-0286-z (PMC6582526; doi:10.1186/s13034-019-0286-z)
Supplement: Supplementary file 8 — Additional file 8. Comparison of OCD-CA parent ratings in the OCDS and non-OCD in children aged 6 to 10 years old. OCD-CA parent ratings of the 6 to 10 years old children in the OCD subsample and the non-OCD clinical subsample (patients with other psychological disorders) are compared. [file 13034_2019_286_MOESM8_ESM.pdf]

Additional file 8

Comparison of OCD-CA parent ratings in the OCDS and non-OCD in children aged 6 to 10 years old

**Scale**	**OCDS**	**Non-OCD**	
	M (SD)	*M (SD)*	*t*
Contamination & Washing	8.37 (9.04)	1.98 (3.43)	4.56**
Catastrophes & Injuries	8.33 (8.96)	2.20 (3.03)	4.46**
Checking	3.50 (5.05)	0.70 (1.35)	3.66**
Ordering & Repeating	5.80 (5.12)	1.22 (2.41)	5.64**
OCD Total	29.63 (21.62)	8.17 (8.15)	6.41**

Note: OCDS: n=46, Non-OCD: n=64; **p<=.001
