# Supplementary material for: Psychometric evaluation of a parent-rating and self-rating inventory for pediatric obsessive-compulsive disorder: German OCD Inventory for Children and Adolescents (OCD-CA)
Source: Child Adolesc Psychiatry Ment Health. 2019 Jun 18;13:25. doi: 10.1186/s13034-019-0286-z (PMC6582526; doi:10.1186/s13034-019-0286-z)
Supplement: Supplementary file 9 — Additional file 9. Comparison of means between self- and parent-report form. In the OCD subsample and the community sample self-rated and parent-rated OCD-CA mean scale scores are compared. [file 13034_2019_286_MOESM9_ESM.pdf]

Additional file 9
Comparison of means between self- and parent-report form

Scale	OCD-CA		
	**Self-report** *M (SD)*	**Parent report** *M (SD)*	*t*
Contamination & Washing	9.96 (8.40)	12.86 (10.90)	-4.01**
	(5.54 (4.77))	(2.89 (4.05))	(-9.47**)
Catastrophes & Injuries	9.72 (9.19)	8.98 (8.42)	0.91
	(5.49 (5.65))	(1.94 (3.50))	(-11.87**)
Checking	5.54 (5.43)	4.19 (5.12)	3.09*
	(4.59 (4.03))	(1.43 (2.54))	(-15.11**)
Ordering & Repeating	5.56 (4.50)	6.01 (5.24)	-0.99
	(1.51 (2.08))	(0.65 (1.67))	(-8.28**)
OCD Total	34.31 (23.26)	35.43 (20.56)	-0.56
	(19.39 (14.83))	(8.16 (11.01))	(-13.98**)

Note: OCDS: n=134; (COS: n=367); *p<.01, **p<.001
