# Supplementary material for: Psychometric evaluation of a parent-rating and self-rating inventory for pediatric obsessive-compulsive disorder: German OCD Inventory for Children and Adolescents (OCD-CA)
Source: Child Adolesc Psychiatry Ment Health. 2019 Jun 18;13:25. doi: 10.1186/s13034-019-0286-z (PMC6582526; doi:10.1186/s13034-019-0286-z)
Supplement: Supplementary file 10 — Additional file 10. CLIN: Comparison of means between age groups and gender in the parent form (ANOVA). Results of ANOVA in the combined clinical sample regarding comparison of means between age groups (6–10 years old and 11–18 years old) and gender in the parent form are presented. [file 13034_2019_286_MOESM10_ESM.pdf]

Additional file 10

CLIN: Comparison of means between age groups and gender in the parent form (ANOVA)

Scale	6-10 years old	N	M (SD)	11-18 years old N	M (SD)	Age effect F	Gender effect F	Interaction F
Contamination & Washing	*Overall*	110	4.65 (7.11)	232	8.86 (10.33)			
	Males	72	4.57 (7.50)	134	8.33 (10.79)	14.15**	0.44	0.20
	Females	38	4.82 (6.40)	98	9.59 (9.67)			
Catastrophes & Injuries	*Overall*	110	4.76 (6.90)	232	6.57 (8.03)			
	Males	72	4.57 (7.17)	134	5.51 (7.64)	4.36*	2.79	1.12
	Females	38	5.13 (6.43)	98	8.02 (8.34)			
Checking	*Overall*	110	1.87 (3.68)	232	3.00 (4.45)			
	Males	72	1.56 (3.26)	134	2.48 (4.13)	4.51*	4.48*	0.09
	Females	38	2.47 (4.34)	98	3.69 (4.79)			
Ordering & Repeating	*Overall*	110	3.14 (4.40)	232	3.94 (4.98)			
	Males	72	2.89 (4.66)	134	3.47 (4.78)	1.85	2.55	0.12
	Females	38	3.61 (3.87)	98	4.59 (5.19)			
OCD Total	*Overall*	110	17.15 (18.56)	232	25.04 (22.70)			
	Males	72	16.17 (20.36)	134	22.13 (23.53)	9.72**	3.60	0.63
	Females	38	19.00 (14.61)	98	29.03 (20.99)			

Note: age groups: 6-10 years old and 11-18 years old; *p<.05, **p<.01
