# Supplementary material for: Psychometric evaluation of a parent-rating and self-rating inventory for pediatric obsessive-compulsive disorder: German OCD Inventory for Children and Adolescents (OCD-CA)
Source: Child Adolesc Psychiatry Ment Health. 2019 Jun 18;13:25. doi: 10.1186/s13034-019-0286-z (PMC6582526; doi:10.1186/s13034-019-0286-z)
Supplement: Supplementary file 11 — Additional file 11. OCDS: Comparison of means between age groups and gender in the parent form (ANOVA). Results of ANOVA in the OCD subsample regarding comparison of means between age groups (6–10 years old and 11–18 years old) and gender in the parent form are presented. [file 13034_2019_286_MOESM11_ESM.pdf]

Additional file 11

OCDS: Comparison of means between age groups and gender in the parent form (ANOVA)

Scale	6-10 years old	N	M (SD)	11-18 years old	N	M (SD)	Age effect F	Gender effect F	Interaction F
Contamination & Washing	*Overall*	46	8.37 (9.04)	*Overall*	135	13.06 (10.91)			
	Males	25	9.84 (10.29)	*Males*	66	14.15 (11.68)	7.35*	2.24	0.09
	Females	21	6.62 (7.14)	*Females*	69	12.01 (10.10)			
Catastrophes & Injuries	*Overall*	46	8.33 (8.96)	*Overall*	135	9.28 (8.53)			
	Males	25	9.76 (9.93)	*Males*	66	8.79 (7.89)	0.53	0.54	1.93
	Females	21	6.62 (7.52)	*Females*	69	9.75 (9.13)			
Checking	*Overall*	46	3.50 (5.05)	*Overall*	135	4.36 (5.08)			
	Males	25	3.04 (4.92)	*Males*	66	3.79 (4.97)	0.85	1.49	0.00
	Females	21	4.05 (5.27)	*Females*	69	4.90 (5.16)			
Ordering & Repeating	*Overall*	46	5.80 (5.12)	*Overall*	135	6.10 (5.32)			
	Males	25	6.08 (5.95)	*Males*	66	5.85 (5.46)	0.12	0.00	0.36
	Females	21	5.48 (4.04)	*Females*	69	6.33 (5.21)			
OCD Total	*Overall*	46	29.63 (21.62)	*Overall*	135	36.30 (20.70)			
	Males	25	32.60 (26.48)	*Males*	66	35.77 (21.69)	3.73	0.58	1.10
	Females	21	26.10 (13.64)	*Females*	69	36.80 (19.96)			

Note: age groups: 6-10 years old and 11-18 years old; **p<.05
