# Supplementary material for: Psychometric evaluation of a parent-rating and self-rating inventory for pediatric obsessive-compulsive disorder: German OCD Inventory for Children and Adolescents (OCD-CA)
Source: Child Adolesc Psychiatry Ment Health. 2019 Jun 18;13:25. doi: 10.1186/s13034-019-0286-z (PMC6582526; doi:10.1186/s13034-019-0286-z)
Supplement: Supplementary file 12 — Additional file 12. Comparison of means between boys and girls. Results of ANOVA in the combined clinical sample, OCD subsample and community sample regarding comparison of means between gender in the parent and self-report form are reported. [file 13034_2019_286_MOESM12_ESM.pdf]

Additional file 12
Comparison of means between boys and girls

Scale	Parent form			Self-report form		
	**Boys**	**Girls**		**Boys**	**Girls**	
	M (SD)	*M (SD)*	*t*	*M (SD)*	*M (SD)*	*t*
Contamination & Washing				7.07 (8.16)	8.27 (7.81)	-1.10
				{10.05 (9.14)}	{9.88 (7.69)}	{0.11}
	(2.45 (3.71))	(3.19 (4.25))	(-4.21)	(5.82 (4.98))	(5.36 (4.63))	(0.91)
Catastrophes & Injuries				6.09 (7.43)	9.42 (8.95)	-2.93**
				{8.48 (8.80)}	{10.88 (9.47)}	{-1.52}
	(1.67 (3.05))	(2.12 (3.76))	(-2.23)	(5.58 (5.86))	(5.43 (5.52))	(0.25)
Checking				4.01 (4.95)	4.88 (4.74)	-1.32
				{5.22 (6.07)}	{5.86 (4.77)}	{-0.68}
	(1.28 (2.30))	(1.52 (2.68))	(-2.51)	(4.90 (3.99))	(4.39 (4.05))	(1.20)
Ordering & Repeating				3.34 (3.93)	4.81 (4.72)	-2.45*
				{4.83 (4.20)}	{6.25 (4.70)}	{-1.84}
	(0.45 (1.11))	(0.78 (1.94))	(-1.51*)	(1.74 (2.16))	(1.35 (2.01))	(1.75)
OCD Total				22.89 (22.45)	30.69 (22.38)	-2.55*
				{31.77 (25.02)}	{36.70 (21.39)}	{-1.23}
	(6.98 (9.72))	(8.93 (11.74))	(-3.38)	(20.26 (15.29))	(18.82 (14.52))	(0.91)

Note: CLIN, {OCDS}, (COS); parent form: COS: boys: n=146 girls: n=221; self-report form: CLIN: boys: n=123, girls: n=95; OCD: boys: n=65, girls: n=69; COS: boys: n=146, girls: n=221;
*p<.05, **p<=.01
